# Supplementary material for: Spindle integrity is regulated by a phospho-dependent interaction between the Ndc80 and Dam1 kinetochore complexes
Source: PLoS Genet. 2025 Apr 4;21(4):e1011645. doi: 10.1371/journal.pgen.1011645 (PMC12007717; doi:10.1371/journal.pgen.1011645)
Supplement: S1 Table — (DOCX) [file pgen.1011645.s007.docx]

Supplementary Table 1. Phosphorylation of the budding yeast kinetochore detected by mass spectrometry (putative Mps1 sites **bolded**, putative Ipl1 sites underlined, and putative Cdk1 sites *italicized*).

| **Subcomplex** | **Protein** | **Detected phosphorylation** |
| --- | --- | --- |
| Ndc80 complex | Ndc80 | S37, **T38**, T54, T71, **S73**, T74, S95, **S97**, T248, **T252**, **S487**, **T690** |
|  | Nuf2 | S248, **T374** |
|  | Spc24 | S2, S129, *T130*, S134, **S149**, *T152* |
|  | Spc25 | S100 |
| Dam1 complex | Ask1 | S26, S90, S99, S118, S128, **S134**, **S188**, S200, *S216*, **S244**, *S250*, **S275**, **T276** |
|  | Dad1 | S75, S77, T87, **S91** |
|  | Dad2 |  |
|  | Dad3 | S43, T47 |
|  | Dad4 |  |
|  | Dam1 | S2, S20, S23, S27, S30, S31, T48, S257, S268, S292, S295 |
|  | Duo1 | **S5**, **T30**, **T31**, S43, **S47**, S50, T151 |
|  | Hsk3 |  |
|  | Spc19 | *S116* |
|  | Spc34 | **S192** |
| Spc105 complex | Kre28 | S215 |
|  | Spc105 | **T59**, T65, **S66**, S70, S77, **T83**, S86, T105, S106, **T107**, *S109*, **T111**, S114, S115, T143, **S144**, S158, S168, **T172**, **S250**, **S258**, *S329*, S353, **T355**, *T356*, T364, *S380*, T383, **S385**, **T414**, S445, **T675** |
| Mis12 complex | Dsn1 | S2, **S10**, *T12*, *S69*, S250, *S264*, **T380**, S546, S547, T549, **S554** |
|  | Mtw1 |  |
|  | Nnf1 |  |
|  | Nsl1 | S2, **S165**, T166 |
| CENP-T | Cnn1 | *T3*, *T21*, *T42*, **S105**, *S177*, **S185** |
|  | Wip1 |  |
| OA | Ame1 | *T31*, *S41*, *S45*, *S53*, S59 |
|  | Okp1 | S70, **S117**, S120, **T401** |
| CCAN | Chl4 |  |
|  | Ctf3 |  |
|  | Ctf19 |  |
|  | Iml3 |  |
|  | Mcm16 |  |
|  | Mcm21 | *T88*, T138, *S139* |
|  | Mcm22 |  |
|  | Mif2 | S54, S78, **S88**, S154, *S160*, **S162**, *T166*, T321, **S325** |
|  | Nkp1 | S76 |
|  | Nkp2 |  |
| CENP-A | Cse4 |  |
| SAC | Bub1 | S14, *T566* |
|  | Mps1 | T3, **S5**, **T18**, S22, **T28**, *T29*, S48, **S50**, S54, **S71**, S80, S86, **S92**, T96, S116, **S129**, *S185*, **S281**, **S292**, **S299**, S306, **S311**, **S312**, **S314**, S315, **S329**, **S332**, *S336*, **S363**, S383, T389, S395, **S425**, **T591**, T754 |
| MAPs | Stu2 | S277, *S603*, S813, **S815** |
| CPC | Bir1 | S477, S524, **S640** |
|  | Sli15 | **S70**, **S97**, *S280*, **S421**, *S437*, **S439**, *S489*, S565, *T618* |
| Regulatory | Psh1 | S143, **S191**, T310, S403 |
|  | Ubr2 | *S394*, **S717**, *S1218* |
