# Supplementary material for: Spindle integrity is regulated by a phospho-dependent interaction between the Ndc80 and Dam1 kinetochore complexes
Source: PLoS Genet. 2025 Apr 4;21(4):e1011645. doi: 10.1371/journal.pgen.1011645 (PMC12007717; doi:10.1371/journal.pgen.1011645)
Supplement: S3 Table — (DOCX) [file pgen.1011645.s009.docx]

Supplementary Table 3. Yeast strains used in this study. All strains are derivatives of SBY3 (W303).

| **Strain** | **Relevant Genotype** |
| --- | --- |
| SBY3 (W303) | *MAT*a *ura3-1 leu2-3,112 his3-11 trp1-1 can1-100 ade2-1 bar1-1* |
| SBY293 | *MATa mad3Δ::URA3* |
| SBY8253 | *MATa DSN1-6His-3Flag:URA3* |
| SBY10651 | *MATa NDC80-3Flag:KanMX* |
| SBY12441 | *MATa DAD1-3Flag:TRP1* |
| SBY19699 | *MATa SPC24-6His-3Flag:URA3 leu2::pGPD1-OsTIR1:LEU2 CDC20-IAA7:KanMX* |
| SBY19701 | *MATa SPC24-6His-3Flag:URA3 cdc15-2* |
| SBY19721 | *MATa NDC80-3Flag:KanMX ura3::pGAL-MPS1-Myc:URA3* |
| SBY20502 | *MATa NDC80-3Flag:KanMX mps1-1* |
| SBY20761 | *MATa DAD1-3Flag:TRP1 ura3::pGAL-MPS1-Myc:URA3* |
| SBY21171 | *MATa NDC80-3Flag:KanMX CDC20-IAA7:KanMX leu2::pGPD1-OsTIR1:LEU2* |
| SBY21287 | *MATa DSN1-6His-3Flag:URA3 ndc80Δ::NATMx trp1::pNDC80-ndc80-T248A-T252A:TRP1* |
| SBY21289 | *MATa DSN1-6His-3Flag:URA3 ndc80Δ::NATMx trp1::pNDC80-ndc80-T248D-T252D:TRP1* |
| SBY21299 | *MATa SPC24-6His-3Flag:URA3 ndc80Δ::NATMx trp1::pNDC80-NDC80:TRP1* |
| SBY21301 | *MATa SPC24-6His-3Flag:URA3 ndc80Δ::NATMx trp1::pNDC80-ndc80-T248A-T252A:TRP1* |
| SBY21353 | *MATa DSN1-6His-3Flag:URA3 ndc80Δ::NATMx trp1::pNDC80-NDC80:TRP1* |
| SBY21544 | *MATa NDC80-3Flag:KanMX cdc15-2* |
| SBY21633 | *MATa NDC80:HPHMx* |
| SBY21725 | *MATa ndc80-T248A-T252A:HPHMx* |
| SBY21768 | *MATa ndc80-T248D-T252D:HPHMx* |
| SBY22006 | *MATa NDC80:HPHMx DSN1-6His-3Flag:URA3* |
| SBY22007 | *MATa ndc80-T248A-T252A:HPHMx DSN1-6His-3Flag:URA3* |
| SBY22008 | *MATa ndc80-T248D-T252D:HPHMx DSN1-6His-3Flag:URA3* |
| SBY22011 | *MATa DSN1-6His-3Flag:URA3 ndc80-T248D-T252D:HPHMx cdc15-2* |
| SBY22028 | *MATa NDC80:HPHMx PDS1-18Myc:LEU2* |
| SBY22029 | *MATa ndc80-T248D-T252D:HPHMx PDS1-18Myc:LEU2* |
| SBY22030 | *MATa ndc80-T248A-T252A:HPHMx PDS1-18Myc:LEU2* |
| SBY22062 | *MATa DSN1-6His-3Flag:URA3 NDC80:HPHMx cdc15-2* |
| SBY22063 | *MATa DSN1-6His-3Flag:URA3 ndc80-T248A-T252A:HPHMx cdc15-2* |
| SBY22551 | *MATa SPC24-6His-3Flag:URA3 NDC80:HPHMx cdc15-2* |
| SBY22554 | *MATa SPC24-6His-3Flag:URA3 ndc80-T248A-T252A:HPHMx cdc15-2* |
| SBY22557 | *MATa SPC24-6His-3Flag:URA3 ndc80-T248D-T252D:HPHMx cdc15-2* |
| SBY22730 | *MATa NDC80:HPHMx ask1-2* |
| SBY22732 | *MATa ndc80-T248A-T252A:HPHMx ask1-2* |
| SBY22734 | *MATa ndc80-T248D-T252D:HPHMx ask1-2* |
| SBY22736 | *MATa NDC80:HPHMx dad1-1:KanMX* |
| SBY22738 | *MATa ndc80-T248A-T252A:HPHMx dad1-1:KanMX* |
| SBY22740 | *MATa ndc80-T248D-T252D:HPHMx dad1-1:KanMX* |
| SBY22771 | *MATa MTW1-mKate2:KanMX leu2::GFP-TUB1:LEU2* |
| SBY22852 | *MATa MTW1-mKate2:KanMX leu2::GFP-TUB1:LEU2 ask1-2 NDC80:HPHMx* |
| SBY22853 | *MATa MTW1-mKate2:KanMX leu2::GFP-TUB1:LEU2 ask1-2 ndc80-T248A-T252A:HPHMx* |
| SBY22854 | *MATa MTW1-mKate2:KanMX leu2::GFP-TUB1:LEU2 ask1-2 ndc80-T248D-T252D:HPHMx* |
| SBY23116 | *MATa leu2::GFP-TUB1:LEU2 DAD1-mKate2:KanMX* |
| SBY23144 | *MATα leu2::GFP-TUB1:LEU2 DAD1-mKate2:KanMX NDC80:HPHMx* |
| SBY23145 | *MATα leu2::GFP-TUB1:LEU2 DAD1-mKate2:KanMX ndc80-T248A-T252A:HPHMx* |
| SBY23146 | *MATα leu2::GFP-TUB1:LEU2 DAD1-mKate2:KanMX ndc80-T248D-T252D:HPHMx* |
| SBY23361 | *MATa DAD1-GFP:KanMX MTW1-mCherry:KanMX NDC80:HPHMx* |
| SBY23380 | *MATa DAD1-GFP:KanMX MTW1-mCherry:KanMX ndc80-T248A-T252A:HPHMx* |
| SBY23385 | *MATa DAD1-GFP:KanMX MTW1-mCherry:KanMX ndc80-T248D-T252D:HPHMx* |
| SBY23417 | *MATa DAD1-GFP:KanMX MTW1-mKate2:NATMx* |
| SBY23673 | *MATa DAD1-GFP:KanMX NDC10-mCherry:HPHMx NDC80:HPHMx* |
| SBY23675 | *MATa DAD1-GFP:KanMX NDC10-mCherry:HPHMx ndc80-T248A-T252A:HPHMx* |
| SBY23677 | *MATa DAD1-GFP:KanMX NDC10-mCherry:HPHMx ndc80-T248D-T252D:HPHMx* |
| SBY24184 | *MATa ask1-2 mad3Δ::URA3 ura3::pCUP-LacI-GFP:URA3 chr8::CEN-LacO:TRP1 NDC80:HPHMx* |
| SBY24185 | *MATa ask1-2 mad3Δ::URA3 ura3::pCUP-LacI-GFP:URA3 chr8::CEN-LacO:TRP1 ndc80-T248A-T252A:HPHMx* |
| SBY24186 | *MATa ask1-2 mad3Δ::URA3 ura3::pCUP-LacI-GFP:URA3 chr8::CEN-LacO:TRP1 ndc80-T248D-T252D:HPHMx* |
| SBY24251 | *MATa NDC80-3Flag:KanMX CDC20-IAA7:KanMX KAR3-3V5-IAA7:KanMX leu2::pGPD1-OsTIR1:LEU2* |
| SBY24257 | *MATa ask1-2 ndc80-T248A-T252A:HPHMx mad3Δ::URA3* |
| SBY24281 | *MATa SPC24-6His-3Flag:URA3 NDC80:HPHMx cdc15-2 ndc10-1* |
| SBY24282 | *MATa SPC24-6His-3Flag:URA3 ndc80-T248D-T252D:HPHMx cdc15-2 ndc10-1* |
| SBY24287 | *MATa MTW1-mKate2:KanMX NUF2-mGFP:KanMX NDC80:HPHMx* |
| SBY24288 | *MATa MTW1-mKate2:KanMX NUF2-mGFP:KanMX ndc80-T248A-T252A:HPHMx:HPHMx* |
| SBY24289 | *MATa MTW1-mKate2:KanMX NUF2-mGFP:KanMX ndc80-T248D-T252D:HPHMx* |
| SBY24290 | *MATa mad3Δ::URA3 ura3::pCUP-LacI-GFP:URA3 chr8::CEN-LacO:TRP1* |
