# Supplementary material for: Spindle integrity is regulated by a phospho-dependent interaction between the Ndc80 and Dam1 kinetochore complexes
Source: PLoS Genet. 2025 Apr 4;21(4):e1011645. doi: 10.1371/journal.pgen.1011645 (PMC12007717; doi:10.1371/journal.pgen.1011645)
Supplement: S4 Table — (DOCX) [file pgen.1011645.s010.docx]

Supplementary Table 4. Plasmids used in this study.

| **Plasmid #** | **Purpose** |
| --- | --- |
| pSB340 | Integration of *GFP-TUB1* at the *LEU2* locus |
| pSB2933 | pFA6a-mKATE2-KAN plasmid for tagging yeast proteins (Gift from Elçin Ünal, University of California, Berkeley) |
| pSB3218 | URA-CEN-sgRNA-Cas9 plasmid (Gift from Elçin Ünal) |
| pSB3348 | Single copy integration of *pNDC80-NDC80* at the *TRP1* locus |
| pSB3349 | Single copy integration of *pNDC80-ndc80^T248A,T252A^* at the *TRP1* locus |
| pSB3350 | Single copy integration of *pNDC80-ndc80^T248D,T252D^* at the *TRP1* locus |
| pSB3408 | URA-CEN-sgRNA-Cas9 plasmid targeting GAGCCAGCCTTTAAAGACTT for *ndc80^T248A,T252A^* and *ndc80^T248D,T252D^* construction |
| pSB3512 | pFA6a-link-mGFP(EGFP^A206K^)-KAN plasmid for tagging yeast proteins (Gift from Elçin Ünal) |
