# Supplementary material for: Spindle integrity is regulated by a phospho-dependent interaction between the Ndc80 and Dam1 kinetochore complexes
Source: PLoS Genet. 2025 Apr 4;21(4):e1011645. doi: 10.1371/journal.pgen.1011645 (PMC12007717; doi:10.1371/journal.pgen.1011645)
Supplement: S5 Table — (DOCX) [file pgen.1011645.s011.docx]

Supplementary Table 5. Oligonucleotides used in this study. All primers are listed 5’ to 3’.

| **Oligo #** | **Purpose** | **Sequence** |
| --- | --- | --- |
| SB6842 | To make *MTW1-mKate2* via homologous recombination (forward) | TATTGAAGAGCCTCAATTGGATTTACTTGATGATGTGTTAggtcgacggatccccgggtt |
| SB392 | To make *MTW1-mKate2* via homologous recombination (reverse) | ATACATCATATCATAGCACATACTTTTTCCCACTTTATATCGATGAATTCGAGCTCGTT |
| SB7295 | To make *DAD1-mKate2* via homologous recombination (forward) | AGTTTATCAGACGAAGCGCCCATCGACGAGCAACCTACTTTATCTCAATCGAAAACGAAGcggatccccgggttaattaa |
| SB7296 | To make *DAD1-mKate2* via homologous recombination (reverse) | ATTTGCGACTGTAAACATAAATTTAGGATAATATTAGGAGAGACAGAGGGAACCGCAACTgaattcgagctcgtttaaac |
| SB7731 | To insert marker 200bp downstream of *NDC80* stop codon (forward) | AATGTTCTATTTTTTTTTTATTTTTTATATTATGGAAGAAAATGGACGTGACGAATCAATcggatccccgggttaattaa |
|  | To insert marker 200bp downstream of *NDC80* stop codon (reverse) | GGTTTCATTGTCACCTTCCTATTTTGAATCTATTCAGTCATAATACCTTTTTGTTCCCACgaattcgagctcgtttaaac |
| SB7757 | To screen *ndc80* T248/T252 mutants (forward) | GTCTATCGAGAATGAGATCTATC |
| SB7758 | To screen *ndc80* T248/T252 mutants (reverse) | CTTTAGTTCTTGCATCGAAGGC |
| SB7753 | To clone *NDC80* sgRNA into Cas9 plasmid: pSB3218 (forward) | ggctgggcaacaccttcgggtggcgaatggGAGCCAGCCTTTAAAGACTT |
| SB7754 | To clone *NDC80* sgRNA into Cas9 plasmid: pSB3218 (reverse) | attttaacttgctatttctagctctaaaacAAGTCTTTAAAGGCTGGCTC |
| SB7810 | CRISPR repair template for *ndc80^T248A,T252A^* | CAAATTTCTTGGCATGTTGCATTGGATGGTACGAACAAATATTAAACTGGATATGTGCTTGAATAAAGTAGATCGTTCATTGATTAATCAAAATGCACAAGAAATAGCAATTCTGAGCCAGCCTTTAAAGACTTTAGACGAACAGGACCAAAGACAAGAAAGATATGAGCTAATGGTGGAGAAACTGTTAATTGATTATT |
| SB7811 | CRISPR repair template for *ndc80^T248D,T252D^* | CAAATTTCTTGGCATGTTGCATTGGATGGTACGAACAAATATTAAACTGGATATGTGCTTGAATAAAGTAGATCGTTCATTGATTAATCAAAATGACCAAGAAATAGACATTCTGAGCCAGCCTTTAAAGACTTTAGACGAACAGGACCAAAGACAAGAAAGATATGAGCTAATGGTGGAGAAACTGTTAATTGATTATT |
| SB9049 | To make *NUF2-mGFP* via homologous recombination (forward) | TTTATTATCTGGTCATATTAATAAATACATGAATGAAATGCTCGAATATATGCAAggtgacggtgctggttta |
| SB9050 | To make *NUF2-mGFP* via homologous recombination (reverse) | AAAAAAAAAAAAAGAAGAAAACACAGAAGGGGGAGTAAAAATAAGTATACCGCTGtcgatgaattcgagctcg |
