# Supplementary material for: Spindle integrity is regulated by a phospho-dependent interaction between the Ndc80 and Dam1 kinetochore complexes
Source: PLoS Genet. 2025 Apr 4;21(4):e1011645. doi: 10.1371/journal.pgen.1011645 (PMC12007717; doi:10.1371/journal.pgen.1011645)
Supplement: Attachment — Submitted filename: Review_Biggins_revision(3).docx [file pgen.1011645.s013.docx]

Spindle integrity is regulated by a phospho-dependent interaction

between the Ndc80 and Dam1 kinetochore complexes" (PGENETICS-D-24-01251R1)

The authors have addressed my remaining questions with a careful revision. I am fully supportive of publication.

1. They provide evidence that the interaction can be requlated in the absence of kinetochores, using a kinetochore-null ndc10-1 allele. (Figure 5E).

2. The conducted additional microscopy experiment, testing the localization of Ndc80c to anaphase spindles. (Supplementary Figure 3).

3. they provide better explanations for the hypomorphic nature of GFP-Tub1 effects.

I would still suggest to optimize display of the location of the mutant residues in Figure 2. Below a quick suggestion using pdb 5TCS (Ndc80c dwarf) The superposition with the human structure (2ve7) could be omitted as the position of Hec1 S202 is not quite the same.
