# Supplementary material for: Spindle integrity is regulated by a phospho-dependent interaction between the Ndc80 and Dam1 kinetochore complexes
Source: PLoS Genet. 2025 Apr 4;21(4):e1011645. doi: 10.1371/journal.pgen.1011645 (PMC12007717; doi:10.1371/journal.pgen.1011645)
Supplement: Attachment — Submitted filename: response_to_reviewers.docx [file pgen.1011645.s014.docx]

We are grateful to the reviewers for their thoughtful comments and have addressed them point by point below:

Reviewer #1: In their paper titled “Spindle integrity is regulated by a phospho-dependent interaction between the Ndc80 and Dam1 kinetochore complexes”, authors Nelson, Mallett, and Biggins examine the yeast kinetochore phosphoproteome and mechanistically dissect the function of two previously uncharacterized phosphosites on the kinetochore protein Ndc80. They first used mass spectrometry on purified kinetochores and identified many previously unidentified phosphorylation sites. Many of these sites fit the consensus sequences for the main mitotic kinases: Ipl1, Mps1, and Cdc28. Mps1 mutants cannot accurately biorient chromosomes in metaphase. However, none of the kinase’s substrate’s phosphosite mutants have fully recapitulated this phenotype, leading the authors to explore the novel Mps1 targets in the hopes of identifying biorientation mutants. They focus on two phosphosites in the MT attachment protein Ndc80. The authors first show that these sites are phosphorylated by Mps1 in vitro and in vivo. They then characterize the phenotype of non-phosphorylatable and phosphmimic mutations for the two sites. They find that there is not effect on chromosome segregation. However, there is some change to Dam1 complex localization and the non-phosphorylatable mutants are synthetic lethal with hypomorphic mutations in the Dam1 complex. This combination of mutants leads to some defects in spindle morphology in metaphase and anaphase. They conclude that a soluble pool of Ndc80 regulates Dam1 function at the mitotic spindle.

Overall, the thorough identification of phosphosites in the yeast kinetochore provides a valuable resource to the community. While some of the phenotypes that they find for the two Ndc80 sites are intriguing, the evidence for their model of soluble pools of Ndc80 regulating Dam1 complex localization is not fully founded.

Major issues:
1. The authors find that sensitization with hypomorphic Dam1c mutants leads to growth defects and/or lethality in the T248A,T252A background. This is a clear result that could potentially provide a strong clue to the role of the phosphorylation. However, it is not clear to me how a slight increase in the percentage of spindles with morphological defects would lead to such a phenotype. Do these morphologic changes somehow lead to cell arrest and/or death? It would be worth revisiting potential kinetochore functions in this background. Is there frequent chromosome missegregation with these combinations of mutants? Does the checkpoint get activated?

We have expanded our phenotypic analysis of the double mutant to address the underlying mitotic defects. First, we analyzed cell cycle progression using a budding index assay and found that the double mutant stalls in mitosis due to activation of the spindle assembly checkpoint (Figure 7B). This suggests that the double mutant does have a defect in kinetochore-microtubule attachments as suggested by the reviewer, so we directly analyzed chromosome segregation. We found that the double mutant does have an increase in chromosome loss (Figure 7E). Taken together, these data suggest that the aberrant spindles likely make it harder to make proper kinetochore-microtubule attachments in the double mutant cells. We have added this data to the revised manuscript.

2. On a related note, does the Ndc80 T248A,T252A mutant alone affect spindle morphology or is this only observed in the context of the adk1-2 allele? This information would be useful in determining if the spindle morphology phenotypes correlate with the growth defects. Such morphological changes could also impact the intensity measurements for Dad1 on the spindle (Figure 6D).

We did not observe an obvious change in spindle morphology in the  *ndc80T^248A,T252A^* mutant alone, consistent with the lack of a growth defect in this strain. We have added representative images for each genotype to Figure 6C to clarify this point.

3. Spindle localization intensity for MAPs can vary greatly depending on spindle length in anaphase. For figure 6D, spindle length should be measured to show that it does not change between the measured spindles for the different mutants or it should be made clear that only anaphase spindles within a narrow range of lengths were used for quantification.

To address this point, we have now quantified spindle lengths for the measurements we reported in Figure 6D and provided them in Supplementary Figure 4E. We did not find any significant change in length between the three genotypes, so the altered Dam1 spindle localization is unlikely due to changes in spindle lengths.

4. I do not know of any evidence for the soluble forms of Ndc80c and Dam1c interacting. To propose this as a model, they should show this interaction either with purified complexes or with pull-downs from yeast without kinetochores.

To address this point, we have repeated the Ndc80 IP to look at Dam1 association in the presence and absence of kinetochores. We used the *ndc10-1* mutant that completely abolishes kinetochore assembly and found that that majority of Nd80 still interacts with Dam1 and that this interaction is enhanced in the Ndc80 phosphomimetic. We have added this new data to the paper (Figure 5E) and are grateful for this idea from the reviewer.

5. In figure 3B, the degree of saturation for the different antibodies in the western blots is quite variable. The increase for pT74 and pT54 phosphorylation is subtle, so the oversaturation with the pT248 antibody may be obfuscating a similar increase. A lower exposure time for the pT248 antibody should be shown.

We have rerun the samples for these blots to obtain better exposure times (Figure 3B). We note that the signal from the pT248 antibody is significantly higher than with the other phospho-antibodies, consistent with a larger fraction of Ndc80 having this modification (Supplemental Table 5).

Minor issues:

6. Just because none of the identified Mps1 phosphorylation sites has yet to recapitulate the biorientation issue, that does not necessarily mean that the correct sites have not been found. It could simply mean that there is a lot of redundancy between them, and one would need to mutate most of them in the same strain to get the biorientation phenotype. The authors could consider mentioning this as an alternative theory if they agree.

This is a good point, and we have added it to the Discussion (Lines 448-451).

7. The benomyl dilution series in Figure 4A is missing a positive control.

We thank the reviewer for noticing this and have added a *mad3Δ* control for the benomyl plate in Figure 4A.

Reviewer #2: This manuscript describes a series of experiments analysing phosphor-regulation of kinetochore complexes, in particular Mps1-dependent regulation of Ndc80 and Dam1C. Novel phenotypes are observed showing that phosphorylation of Ndc80 on T248 and T252 control its interaction with Dam1C. Interestingly these point to a function of Dam1c in organising and stabilising the anaphase spindle. The data is of excellent quality and is very nicely and logically presented. I am happy to recommend this manuscript for publication once minor changes have been made.

Lines 126-7. It is worth pointing out that these cells will be metaphase-arrested.

We have added the point that cells were arrested in metaphase to the text (Line 129).

Lines 139-140 (SFig 1E). Upon Mps1 overexpression, lots of MS hits with the actin cytoskeleton were observed. More discussion of this would be interesting.

We appreciate the reviewer’s interest in the surprising result that we obtained many actin cytoskeleton hits on our MS when Mps1 was overexpressed. We did not add further discussion to the text because we do not yet have an easy way to determine whether this is a meaningful observation or is instead an artifact of heavily phosphorylating the proteins and lysing cells to create nonspecific interactions. In the future, we plan to design experiments to determine if this result is a specific interaction and will then pursue the possible function if we can confirm its specificity.

Line 201. Were Abs to T252 also attempted to be generated? Can the authors tell if both Thr residues are modified on a single polypeptide, or is it usually one or the other? What ratio?

The residues are both on the same polypeptide and we frequently detected peptides where both residues are phosphorylated (Supplemental Table 5). We did not attempt to make antibodies that recognize T252. However, in the future, it will be important to determine if both residues are phosphorylated together in the same phase of the cell cycle.

Fig 3B. Are there ways to specifically induce error-correction activity (rather than use nocodazole) in Fig3B?

We thank the reviewer for suggesting this interesting experiment. To test this, we used a *kar3-AID* mutant that is known induce bi-orientation defects by creating syntelic attachments. We found that the Ndc80 T248 phosphorylation was not stimulated by this defect, consistent with the nocodazole results. We have added this new data as Figure 3C.

Lines 272-4 At times residue T252 is somewhat ignored in the authors’ description/discussion.

We did not have a way to analyze T252 phosphorylation directly, so we had to be careful about how we described the conclusion about phosphorylation in some experiments. We did not mean to ignore it and were mainly being careful not to make conclusions about a site that we could not directly study in some experiments.

Fig 5C. Please add representative images of the A and D mutants

We have added images for the Ndc80 phosphomutants to Figure 5C.

Fig 6B. Perhaps a 3rd repeat of this experiment would generate significant numbers for the alanine mutant too?

We did not repeat the fraction of spindles with Dad1 localization in the alanine mutant, but we did analyze the total levels of Dad1 on spindles in figure 6D three times. Since it wasn’t clear to us that an increase in the fraction of spindles with Dad1 would enhance our understanding of the phenotype, we directed our time to other revision experiments.

SFig 4. Why is the GFP-tubulin intensity reduced in D mutant? More discussion needed on this. Has it been observed in related, relevant mutants?

We did not further discuss the reduced GFP-tubulin intensity because this is another observation that we do not have additional information to speculate on. Tagging GFP-Tub1 at the endogenous locus is not fully functional and all yeast publications using it maintain it as an ectopic copy rather than an endogenous tagged gene. Our assumption is that the tag affects tubulin function and creates unstable microtubules so cells might preferentially use the endogenous tubulin. We have added the information that this is an ectopic copy to the text to avoid confusion (Lines 364-365, 495-496).

Line 338 should read Supplementary Fig 4B-D.

We have fixed this and appreciate the reviewer noticing the error.

Line 353. Can an alternative phrase be used, replacing ‘nearly dead’?

This is a good point, and we have changed the phrase to “very slow growing”.

Line 427-9. More speculation on bundling/oligomerisation functions of Dam1C would be interesting.

We did not speculate on these functions of Dam1c since we have no data about whether these functions are required for anaphase spindle function and the discussion is reasonably lengthy already.

Line 443. Has Dam1C been purified from anaphase cells to look for spindle interactors?

We are not aware that Dam1c has been purified from anaphase cells to look for interacting proteins on spindles. The Damc1 binds directly to microtubules so we did not consider this point, but it is an interesting question whether there are associated proteins that are involved.

Reviewer #3: Nelson et al address the question how phosphorylation at kinetochores ensures faithful chromosome segregation. They conducted mass-spectrometry experiments on purified kinetochore particles to identify phosphorylated residues at the kinetochore, particularly after overexpression of the Mps1 kinase. They focus on the Ndc80 residues T248 and T252, phosphorylated by Mps1 in metaphase. They characterize phospho-null ndc80 T248A T252A and phosphomimetic T248D T252D mutants and find that the alleles do not impair growth, mitotic progression, or kinetochore composition in a wildtype background. They identify ndc80 T248D T252D to show a higher affinity for the Dam1 complex in anaphase, which correlates with decreased Dam1 content on anaphase spindles. In combination with ts alleles of the Dam1 complex subunits Dad1 and Ask1, Ndc80 T248A T252A shows growth defects and alterations in spindle architecture both in metaphase and anaphase.

Although the characterized ndc80 alleles do not have a strong phenotype, this work represents a meaningful addition to open questions concerning Mps1’s contributions to mitotic fidelity. Thus, the work will be of interest to the field. The experiments are conducted on a very high technical level, logically presented and thoughtfully discussed.

I have a couple of points that should be addressed:

Main points:

While the mapping of the phospho-sites and the demonstration of mps1-dependence is very convincing, the model in the end remains somewhat unclear. The question is, where does the phospho-regulation of the Ndc80-Dam1c interaction occur? The phosphorylation sites are derived from kinetochore purifications, so it would be logical to assume that Mps1 phosphorylates kinetochore-bound Ndc80 to promote Dam1 association. The authors state, however, that the characterized Ndc80 alleles regulate a kinetochore-independent Dam1c pool. Do they think the responsible pool of Ndc80c is kinetochore bound?. If yes: how do they imagine the kinetochore bound Ndc80c regulate Dam1c at another localization? If not, where is the Ndc80 pool localized?

This point was also brought up by reviewer one and we do think the major phosphoregulated interaction is the soluble pool of Ndc80 and Dam1. We addressed this in the revised manuscript by purifying Ndc80 from WT vs *ndc10-1* mutant cells which lack kinetochores (Figure 5E). This experiment indicates that the majority of Ndc80 and Dam1 interact in the absence of kinetochores, and we showed that phosphorylation regulates the non-kinetochore bound pool. We appreciate the reviewers asking for this clarification and hope the model is clearer with this new data.

One obvious thing to do is to localize the ndc80 mutant alleles themselves in fluorescence microscopy and analyze kinetochore and spindle association. Maybe ndc80 T248D T252D with increased Dam1 binding could lead to co localization of the complexes on anaphase spindles and this may somehow cause a problem? The authors should test this.

This is an interesting suggestion, and we tested it by localizing Nuf2-mGFP (as a proxy for Ndc80c localization) in the phosphomutants. We did not directly tag the Ndc80 protein to avoid creating additional hypomorphic issues. We did not see Ndc80c localize to anaphase spindles in either phosphomutant and have added this data as Supplementary Figure 3. The lack of Ndc80 localization to spindles is another reason that we proposed the soluble pool of Ndc80/Dam1 regulates the level of Dam1 available to associate with microtubules.

The authors find reduced spindle labelling with GFP-Tub1 in ndc80 T248D T252D mutants. Does this result in spindle phenotypes, e.g. broken spindles? This should be analyzed or stated if not. It would be nice to show respective micrographs. Do the authors think the interaction between Dam1c and Ndc80c regulates tubulin incorporation/dynamics at spindles? The authors should discuss this.

We have added representative micrographs to Figure 6C but did not observe “broken spindles”. We did not further pursue work on spindles in this mutant because the GFP-Tub1 protein is a hypomorph. Our assumption is that the lower levels of GFP-Tub1 are due to the untagged protein incorporating into spindles more easily. We have now made it clear that the fluorescent copy is ectopic. While we do think that the interaction between Dam1c and Ndc80c could regulate tubulin dynamics in some manner, we did not speculate on this point given our lack of data to address it.

Minor point:

Figure 2B: I’d show a slightly larger portion of Ndc80/Nuf2, to put the T248 and T252 sites into a better structural context. Alphafold models should do a good job of predicting this.

We thank the reviewer for this suggestion and attempted to show a larger portion of Ndc80/Nuf2 but by aligning the alpha-fold predictions for Sc Ndc80 with Hs Ndc80. Unfortunately, the sites on both proteins are at the beginning of unstructured regions (this is why we left out T252). This made the structural comparison more difficult to interpret than what we have presented in Figure 2B so we have left the original image.
